# A data-driven acceleration-level scheme for image-based visual servoing of manipulators with unknown structure

**DOI:** 10.3389/fnbot.2024.1380430

**Published:** 2024-03-20

**Authors:** Liuyi Wen, Zhengtai Xie

**Affiliations:** ^1^The State Key Laboratory of Tibetan Intelligent Information Processing and Application, Qinghai Normal University, Xining, China; ^2^School of Arts, Lanzhou University, Lanzhou, China; ^3^School of Information Science and Engineering, Lanzhou University, Lanzhou, China

**Keywords:** recurrent neural network (RNN), image-based visual servoing (IBVS), data-driven technology, acceleration level, learning and control

## Abstract

The research on acceleration-level visual servoing of manipulators is crucial yet insufficient, which restricts the potential application range of visual servoing. To address this issue, this paper proposes a quadratic programming-based acceleration-level image-based visual servoing (AIVS) scheme, which considers joint constraints. Besides, aiming to address the unknown problems in visual servoing systems, a data-driven learning algorithm is proposed to facilitate estimating structural information. Building upon this foundation, a data-driven acceleration-level image-based visual servoing (DAIVS) scheme is proposed, integrating learning and control capabilities. Subsequently, a recurrent neural network (RNN) is developed to tackle the DAIVS scheme, followed by theoretical analyses substantiating its stability. Afterwards, simulations and experiments on a Franka Emika Panda manipulator with eye-in-hand structure and comparisons among the existing methods are provided. The obtained results demonstrate the feasibility and practicality of the proposed schemes and highlight the superior learning and control ability of the proposed RNN. This method is particularly well-suited for visual servoing applications of manipulators with unknown structure.

## 1 Introduction

Robots can accurately perform complex tasks and have become a vital driving force in industrial production (Agarwal and Akella, 2024). Among industrial robots, redundant robots, equipped with multiple degrees of freedom (DOFs), have gained significant recognition and favor due to their exceptional flexibility and automation capabilities (Tang and Zhang, 2022; Zheng et al., 2024). Therefore, numerous control schemes are designed to extend the application range of redundant robots, such as medical services (Zeng et al., 2024) and visual navigation (Wang et al., 2023). Furthermore, in these application scenarios, information on the external environment and the robot's status is acquired from various sensors, especially for the image capture of visual information (Jin et al., 2023). Therefore, unknown situations inevitably exist caused by sensor limitations, environmental variability, and robot modification, which hinder the evolution of robot applications. To address this issue, intelligent algorithms based on data-driven technology are exploited to process the acquired information and convert it into knowledge to drive the regular operation of the robot system (Na et al., 2021; Xie et al., 2022). Yang et al. (2019) construct a robot learning system by improving the adaptive ability of a robot with the information interaction between the robot and environment, which enhances the safety and reliability of robot applications in reality Peng et al. (2023). Li et al. (2019) investigate a model-free control method to cope with the unknown Jacobian problems inside the robot system. On this basis, the dynamic estimation method of robot parameters is researched in the study by Xie and Jin (2023). However, the aforementioned methods primarily operate at the joint velocity level and cannot directly applicable to robots driven by joint acceleration.

As a crucial robot application, visual servoing simulates the bionic system of human eyes, which can obtain information about real objects through optical devices, thus dynamically responding to a visible object. The fundamental task of visual servoing is to impose the error between the corresponding image feature and the desired static reference to approach zero (Zhu et al., 2022). According to the spatial position or image characteristics of the robot, the visual servoing system can be categorized into two types: position-based visual servoing (PBVS) system (Park et al., 2012), which utilizes 3-D position and orientation information to adjust the robot's state, and image-based visual servoing (IBVS) system, which utilizes 2-D image information for guidance (Van et al., 2018). Recently, the research on visual servoing has achieved many unexpected results (Hashimoto et al., 1991; Malis et al., 2010; Zhang et al., 2017; Liang et al., 2018). For instance, visual servoing is applied to bioinspired soft robots in the underwater environment with an adaptive control method, which extends the scope of visual servoing (Xu et al., 2019). Based on the neural network method, a resolution scheme for IBVS is developed at the velocity level. This enables the manipulator to accurately track fixed desired pixels, resulting in fast convergence (Zhang and Li, 2018). However, the aforementioned methods are difficult to deal with the emergence of unknown conditions, such as focal length change, robot abrasion, or parameter variation. This is because these methods rely on accurate structural information of the robot vision system. To tackle this challenge, this study focuses on data-driven control of visual servoing for robots with an unknown Jacobian matrix.

Neural networks have gained significant recognition as powerful tools for solving challenging problems, such as automatic drive (Jin et al., 2024), mechanism control (Xu et al., 2023), and mathematical calculation (Zeng et al., 2003; Stanimirovic et al., 2015). In robot redundancy analysis, neural networks have shown superior performance. In recent decades, numerous control laws based on neural networks have been developed to harness the potential of redundant manipulators (Zhang and Li, 2023). One specific application of the neural network approach addresses the IBVS problem. In this context, the IBVS problem is formulated as a quadratic programming scheme and tackled using a recurrent neural network (RNN). The RNN drives the robot vision system's feature to rapidly converge toward the desired point (Zhang et al., 2017). Additionally, Li et al. (2020) investigate an inverse-free neural network technique to deal with the IBVS task, ensuring that the error approaches zero within a finite time while considering the manipulator's physical constraints.

**Figure 1:**
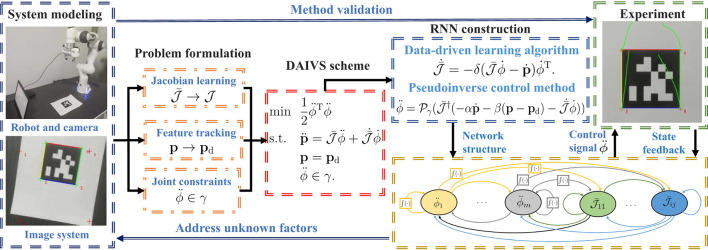
Technical route of this study.

Most control schemes accomplish the given task at the velocity level, especially for visual servoing applications (Hashimoto et al., 1991; Malis et al., 2010; Zhang et al., 2017; Liang et al., 2018; Van et al., 2018; Zhang and Li, 2018; Xu et al., 2019; Li et al., 2020). These velocity-level schemes control redundant robots via joint velocities. However, when confronted with acceleration or torque-driven robots, the velocity-level schemes exhibit limitations and cannot provide precise control. Furthermore, the velocity-level scheme may yield abrupt joint velocities that are impractical in real-world applications. Consequently, research on acceleration-level visual servoing for robot manipulators has become crucial (Keshmiri et al., 2014; Anwar et al., 2019). Motivated by the issues above, this study investigates the application of visual servoing in robots at the acceleration level. The technical route of this study is shown in Figure 1. As illustrated, the contributions of this study are shown as follows:

- An acceleration-level image-based visual servoing (AIVS) scheme is designed, taking into account multiple joint constraints.
- Considering potential unknown factors in the visual servoing system, a data-driven acceleration-level image-based visual servoing (DAIVS) scheme is developed, enabling simultaneous learning and control.
- RNNs are proposed to solve the AIVS scheme and DAIVS scheme, enabling visual servoing control of the manipulator. Theoretical analyses guarantee the stability of the RNNs.

In addition, the feasibility of the proposed schemes is demonstrated through simulative and experimental results conducted on a Franka Emika Panda manipulator with an eye-in-hand structure.

Before concluding this section, the remaining sections of the study are shown as follows. Section 2 presents the robot kinematics of visual servoing and introduces the data-driven learning algorithm, formulating the problem at the acceleration level. Section 3 constructs an AIVS scheme with the relevant RNN. Subsequently, considering the unknown factors, a DAIVS scheme and corresponding RNN are proposed, and theoretical analyses proved the learning and control ability of the RNN, as shown in Section 4. Section 5 provides abundant simulations and performance comparisons, embodying the proposed method's validity and superiority. Section 6 displays physical experiments on a real manipulator. Finally, Section 7 briefly concludes this study.

## 2 Preliminaries

In this section, the robot visual servoing kinematics and data-driven learning algorithm are introduced as the preliminaries. Note that this study specifically tackles the problem at the acceleration level.

### 2.1 Robot visual servoing kinematics

**Figure 2:**
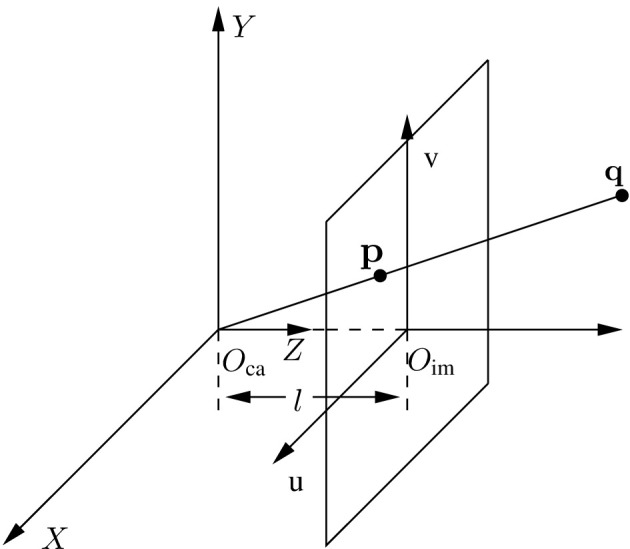
Geometric schematic of the camera system.

The forward kinematics, which contains the transformation between the joint angle ϕ(*t*)∈ℝ^*m*^ of a robot and the end-effector position and posture **s**(*t*)∈ℝ^6^, can be expressed as follows:


(1) $$\begin{array}{l} f\left(\phi \left(t\right)\right)=\text{s}\left(t\right), \end{array}$$

where *f*(·) is the non-linear mapping related to the structure of the robot. In view of strongly non-linear and redundant characteristics of *f*(·), it is difficult to obtain the desired angle information directly from the desired end-effector information **s**_d_(*t*), i.e., **s**(*t*) = **s**_d_(*t*). By taking the time derivative of both sides of Equation (1), one can deduce


(2) $$\begin{array}{l} J_{\text{ro}}\dot{\phi }\left(t\right)=\dot{\text{s}}\left(t\right), \end{array}$$

where $\dot{\phi }\left(t\right)$ denotes the joint velocity; $\dot{\text{s}}\left(t\right)$ covers the joint velocity and translational velocity of the end-effector; $J_{\text{ro}}=\partial f\left(\phi \left(t\right)\right)/\partial \phi \left(t\right)\in {\mathbb{R}}^{6\times m}$ stands for the robot Jacobian matrix. Owing to the physical properties of manipulators, output control signals based on design formulas and intelligent calculations may not be suitable for the normal operation of real robots. Therefore, to ensure the protection of the robot, it is crucial to take into account the following joint restrictions:


$$\begin{array}{l} \phi^{-}\leq \phi \leq \phi^{+}\\ {\dot{\phi }}^{-}\leq \dot{\phi }\leq {\dot{\phi }}^{+}\\ {\ddot{\phi }}^{-}\leq \ddot{\phi }\leq {\ddot{\phi }}^{+}, \end{array}$$

where ϕ^−^, ${\dot{\phi }}^{-}$, and ${\ddot{\phi }}^{-}$ signify the lower bounds of joint angle, joint velocity, and joint acceleration, respectively; ϕ^+^, ${\dot{\phi }}^{+}$ and ${\ddot{\phi }}^{+}$ denote the upper bounds of joint angle, joint velocity, and joint acceleration, respectively. Utilizing the special conversion techniques (Zhang and Zhang, 2012; Xie et al., 2022), the joint restrictions would be integrated into the acceleration level as $\ddot{\phi }\in \gamma ,$ where γ = {*g*∈ℝ^*m*^, γ^−^ ≤ *g* ≤ γ^+^} is the safe range of joints with γ^−^ and γ^+^ denoting the lower bound and upper bound of γ, respectively. In detail, the *i*-th elements of γ^−^ and γ^+^ are designed as


$$\begin{array}{l} \begin{array}{c} \gamma_{i}^{-}=\text{max}\left\{\mu \left(\phi_{i}^{-}+\theta_{i}-\phi_{i}\right),\nu \left({\dot{\phi }}_{i}^{-}-{\dot{\phi }}_{i}\right),{\ddot{\phi }}_{i}^{-}\right\}\\ \gamma_{i}^{+}=\text{min}\left\{\mu \left(\phi_{i}^{+}-\theta_{i}-\phi_{i}\right),\nu \left({\dot{\phi }}_{i}^{+}-{\dot{\phi }}_{i}\right),{\ddot{\phi }}_{i}^{+}\right\}, \end{array} \end{array}$$

where *i* = 1, 2, 3, ⋯ , *m*; μ>0 and ν>0 are designed to select the feasible region for different levels; θ_*i*_ is the margin to ensure that the acceleration has a sufficiently large feasible region (Xie et al., 2022). Then, a brief introduction to the visual servoing system is presented as follows. Regarding visual servoing tasks, the number of features determines the complexity of a visual servoing system. Simply considering a visual servoing system with one feature, a miniature camera is mounted on the end-effector of the manipulator and moves with the end-effector. Figure 2 illustrates the geometric transformation in different coordinate systems. Three-dimensional space with *O*_ca_ as the original point and [*X, Y, Z*] as the coordinate axis is called the camera system with the internal coordinate point **q** = [*x, y, z*]^T^. Relatively, with *O*_im_ as the center point, the image system is the two-dimensional space with the projection pixel point of **q** being ${\left[p_{x},p_{y}\right]}^{\text{T}}$ and the pixel coordinate being $\text{p}={\left[p_{u},p_{v}\right]}^{\text{T}}\in {\mathbb{R}}^{2}$. According to the similar triangle, it can be readily obtained in the study by Zhang et al. (2017) and Zhang and Li (2018):


(3) $$\begin{array}{l} \left[\begin{array}{c} p_{x}\\ p_{y} \end{array}\right]=\frac{l}{z}\left[\begin{array}{c} x\\ y \end{array}\right] \end{array}$$

and


(4) $$\begin{array}{l} p_{u}=u_{0}+a_{x}p_{x} \end{array}$$

$$\begin{array}{l} p_{v}=v_{0}+a_{y}p_{y}, \end{array}$$

with *l* standing for the focal length of the camera; *u*_0_ and *v*_0_ denoting the pixel coordinate of principle point; and ${\left[a_{x},a_{y}\right]}^{\text{T}}$ standing for the conversion scale. Based on Equations (3, 4), the image Jacobian matrix $J_{\text{im}}\left(\text{p},z\right)\in {\mathbb{R}}^{2\times 6}$ is defined using the following relationship (Liang et al., 2018):


(5) $$\begin{array}{l} J_{\text{im}}\left(\text{p},z\right)\dot{\text{s}}=\dot{\text{p}}, \end{array}$$

where $\dot{\text{p}}$ stands for the movement velocity of the pixel coordinate and


$$\begin{array}{l} J_{\text{im}}\left(\text{p},z\right)=H\left[\begin{array}{cccccc} -\frac{l}{z} & 0 & \frac{lp_{x}}{z} & \frac{p_{x}p_{y}}{l} & -\frac{p_{x}^{2}+l^{2}}{l} & p_{y}\\ 0 & -\frac{l}{z} & \frac{p_{y}}{z} & -\frac{p_{y}^{2}+l^{2}}{l\;} & -\frac{p_{x}p_{y}}{l} & -p_{x} \end{array}\right], \end{array}$$

with


$$\begin{array}{l} \;p_{x}=\frac{p_{u}-u_{0}}{a_{x}},\;p_{y}=\frac{p_{v}-v_{0}}{a_{y}},\;H=\left[\begin{array}{cc} a_{x} & 0\\ 0 & a_{y} \end{array}\right]. \end{array}$$

For the sake of convenience, Equations (2, 5) can be combined as follows:


(6) $$\begin{array}{l} J\dot{\phi }=\dot{\text{p}}, \end{array}$$

with $J=J_{\text{im}}\left(\text{p},z\right)J_{\text{ro}}\in {\mathbb{R}}^{2\times m}$ defined as the visual Jacobian matrix. The relationship between joint space and image space is established directly by Equation (6) at the velocity level. Taking the time derivatives of both sides of Equation (6) generates


(7) $$\begin{array}{l} \dot{J}\dot{\phi }+J\ddot{\phi }=\ddot{\text{p}}, \end{array}$$

where $\dot{J}$ is the time derivative of 𝒥; $\ddot{\phi }$ denotes the joint acceleration; and $\ddot{\text{p}}$ stands for the movement acceleration of the pixel coordinate. When it comes to a complicated situation with more features, the above analyses still hold under the requirements of appropriate dimensions. It is worth noting that a single feature is analyzed as an example for simple illustration. When the number of features increases, the principle of coordinate transformation remains unchanged along with the increase in dimension.

### 2.2 Data-driven learning algorithm

However, unknown conditions may exist in the robot visual servoing system, such as focal length changes or robot modifications. In this regard, it could not control the robot accurately to execute the IBVS task based on $\dot{J}$. Hence, motivated by this issue, a data-driven learning algorithm is designed as follows. To begin with, a virtual IBVS system is established, incorporating the virtual visual Jacobian matrix $\bar{J}\in {\mathbb{R}}^{2\times m}$ and the following relationship:


$$\begin{array}{l} \bar{J}\dot{\phi }=\bar{\dot{\text{p}}}, \end{array}$$

where $\bar{\dot{\text{p}}}\in {\mathbb{R}}^{2}$ is the virtual pixel velocity determined by the virtual robot and $\dot{\phi }$ is the joint velocity measured in real time from the robot. Beyond dispute, the goal of the data-driven learning algorithm is to guarantee that $\bar{\dot{\text{p}}}$ can rapidly converge to the real pixel velocity $\dot{\text{p}}$. Thereout, an error function is devised as $\ell =||\bar{\dot{\text{p}}}-\dot{\text{p}}|{|}_{2}^{2}/2$, where ||·||_2_ is the Euclidean norm of a vector. On the basis of the gradient descent method (Stanimirovic et al., 2015) to minimize the error function along the negative gradient direction, one can get


(8) $$\begin{array}{l} \dot{\bar{J}}=-\delta \frac{\partial \ell }{\partial \bar{J}}=-\delta \left(\bar{J}\dot{\phi }-\dot{\text{p}}\right){\dot{\phi }}^{\text{T}}, \end{array}$$

where $\dot{\bar{J}}$ is the time derivation of $\bar{J}$; δ>0 denotes the coefficient that controls the convergence rate. Hereinafter, $\dot{\bar{J}}$ and $\bar{J}$ are used to replace the calibrated parameter $\dot{J}$ and 𝒥 to deal with the unknown situations. This method directly explores the relationship between joint space and image space without the utilization of $\dot{J}$ and 𝒥. It is worth highlighting that Equation (8) does not involve real structural information and estimates structural information from the joint velocity $\dot{\phi }$ and velocity of the pixel coordinate $\dot{\text{p}}$ measured by sensors, which belongs to the core idea of the data-driven learning algorithm.

## 3 Acceleration-level IBVS solution

In this section, an AIVS scheme is proposed with joint constraints considered. Subsequently, we propose a corresponding RNN and provide theoretical analyses. Note that the presented method requires an accurate visual Jacobian matrix.

### 3.1 AIVS scheme

It is worth pointing out that there are few acceleration-level robot control schemes for dealing with IBVS problems. None of the existing acceleration-level solutions take joint constraints into account (Keshmiri et al., 2014; Anwar et al., 2019). In this regard, considering joint constraints, acceleration control, and visual servoing kinematics, the AIVS scheme is constructed as a quadratic programming problem, taking the following form:


(9a) $$\begin{array}{l} \text{minimize }\frac{1}{2}{\ddot{\phi }}^{\text{T}}\ddot{\phi } \end{array}$$

(9b) $$\begin{array}{l} \text{subject to }\ddot{\text{p}}=J\ddot{\phi }+\dot{J}\dot{\phi } \end{array}$$

(9c) $$\begin{array}{l} \;\text{p}={\text{p}}_{\text{d}} \end{array}$$

(9d) $$\begin{array}{l} \;\ddot{\phi }\in \gamma , \end{array}$$

where **p**_d_ denotes the desired pixel coordinate. As a result, the goal of AIVS scheme (9) is to make the end-effector track the desired pixel point. In addition, according to robot Jacobian matrix *J*_ro_ and the image Jacobian matrix *J*_im_, the visual Jacobian matrix 𝒥 and its time derivative $\dot{J}$ are determined by the structure and parameters of the robot and the parameter settings inside the camera. Hence, if there are any changes in the internal parameters or structures, leading to an unknown state, the accuracy of 𝒥 and $\dot{J}$ may be compromised, potentially leading to a decline in performance. In contrast to velocity-level visual servoing schemes (Hashimoto et al., 1991; Malis et al., 2010; Zhang et al., 2017; Liang et al., 2018; Van et al., 2018; Zhang and Li, 2018; Xu et al., 2019; Li et al., 2020), the proposed AIVS scheme (9) offers two advantages. First, it utilizes joint acceleration as the control signal, resulting in continuous joint velocities. This helps mitigate the issues associated with excessive and discontinuous joint velocities. Second, AIVS scheme (9) takes into account the equality and inequality constraints at the acceleration level. This allows for a more comprehensive consideration of constraints, expanding the range of applications.

### 3.2 RNN solution and theoretical analysis

For the AIVS scheme (9), the pseudoinverse method is applied to generate the relevant RNN solution (Cigliano et al., 2015; Li et al., 2020). Primarily, as reported in the study by Zhang and Zhang (2012) and Xie et al. (2022), one can readily extend pixel coordinate error **p**−**p**_d_ into the acceleration level by neural dynamics method (Liufu et al., 2024) as


(10) $$\begin{array}{l} \ddot{\text{p}}-{\ddot{\text{p}}}_{\text{d}}=-\alpha \left(\dot{\text{p}}-{\dot{\text{p}}}_{\text{d}}\right)-\beta \left(\text{p}-{\text{p}}_{\text{d}}\right), \end{array}$$

where the design parameter α>0 and β>0; ${\dot{\text{p}}}_{\text{d}}$ and ${\ddot{\text{p}}}_{\text{d}}$ are the desired velocity and the desired acceleration of the pixel coordinates, respectively. It is worth pointing out that the desired pixel coordinates **p**_d_ is a constant, thus ${\dot{\text{p}}}_{\text{d}}={\ddot{\text{p}}}_{\text{d}}=0$. As a result, Equation (10) can be rearranged as


(11) $$\begin{array}{l} \ddot{\text{p}}=-\alpha \dot{\text{p}}-\beta \left(\text{p}-{\text{p}}_{\text{d}}\right). \end{array}$$

Substituting Equation (11) into Equation (9b), it could be obtained:


$$\begin{array}{l} \dot{J}\dot{\phi }+J\ddot{\phi }=-\alpha \dot{\text{p}}-\beta \left(\text{p}-{\text{p}}_{\text{d}}\right). \end{array}$$

In light of the pseudoinverse method, the joint acceleration can be minimized with the following formula:


(12) $$\begin{array}{l} \ddot{\phi }=J^{†}\left(-\alpha \dot{\text{p}}-\beta \left(\text{p}-{\text{p}}_{\text{d}}\right)-\dot{J}\dot{\phi }\right), \end{array}$$

where superscript ^†^ denotes the pseudoinverse operation of a matrix with 𝒥^†^ = 𝒥^T^(𝒥𝒥^T^)−1. It is deserved to note that Equation (12) is employed in the study by Keshmiri et al. (2014) and Anwar et al. (2019) to generate the acceleration command for a manipulator. However, the research in the study by Keshmiri et al. (2014) and Anwar et al. (2019) does not consider joint constraints of the manipulator. To address this problem, the RNN corresponding to the AIVS scheme (9) is derived as


(13) $$\begin{array}{l} \ddot{\phi }=p_{\gamma }\left(J^{†}\left(-\alpha \dot{\text{p}}-\beta \left(\text{p}-{\text{p}}_{\text{d}}\right)-\dot{J}\dot{\phi }\right)\right), \end{array}$$

where projection function 𝒫_γ_(*c*) = argmin_*b*∈γ_||*b*−*c*||_2_. Furthermore, theoretical analyses regarding the convergence of RNN (13) are presented as follows.

*Theorem 1:* The pixel error ξ = **p**−**p**_d_ driven by AIVS scheme (9) assisted with RNN (13) globally converges to a zero vector.

*Proof:* According to Equations (7, 13), one has


$$\begin{array}{l} \ddot{\text{p}}=J\ddot{\phi }+\dot{J}\dot{\phi }=Jp_{\gamma }\left(J^{†}\left(-\alpha \dot{\text{p}}-\beta \left(\text{p}-{\text{p}}_{\text{d}}\right)-\dot{J}\dot{\phi }\right)\right)+\dot{J}\dot{\phi }. \end{array}$$

Due to the fact that **p**_d_ is a fixed feature, error function $\ddot{\xi }$ can be readily derived as


$$\begin{array}{l} \ddot{\xi }=JP_{\gamma }\left(J^{†}\left(-\alpha \dot{\xi }-\beta \xi -\dot{J}\dot{\phi }\right)\right)+\dot{J}\dot{\phi }. \end{array}$$

By considering the projection function, a substitution matrix H is designed to replace 𝒫_γ_(·), leading to


(14) $$\begin{array}{l} \ddot{\xi }=JHJ^{†}\left(-\alpha \dot{\xi }-\beta \xi -\dot{J}\dot{\phi }\right)+\dot{J}\dot{\phi }, \end{array}$$

of which


$$\begin{array}{l} H=\left[\begin{array}{cccc} h_{1} & 0 & \cdots & 0\\ 0 & h_{2} & \cdots & 0\\ \vdots & \vdots & \ddots & \vdots \\ 0 & 0 & \cdots \; & h_{m} \end{array}\right]\in {\mathbb{R}}^{m\times m} \end{array}$$

and


$$\begin{array}{l} h_{i}=\frac{{\left(P_{\gamma }\left(J^{†}\left(-\alpha \dot{\text{p}}-\beta \left(\text{p}-{\text{p}}_{\text{d}}\right)-\dot{J}\dot{\phi }\right)\right)\right)}_{i}}{{\left(J^{†}\left(-\alpha \dot{\text{p}}-\beta \left(\text{p}-{\text{p}}_{\text{d}}\right)-\dot{J}\dot{\phi }\right)\right)}_{i}}\in \left(0,1\right]. \end{array}$$

By matrix decomposition, structural analyses of matrix $JℋJ^{†}=[a_{11},a_{12};a_{21},a_{22}\in {\mathbb{R}}^{2\times 2}$ are given as follows:


$$\begin{array}{l} JHJ^{†}=JLL^{\text{T}}J^{\text{T}}{\left(JJ^{\text{T}}\right)}^{-1}, \end{array}$$

where $L=\sqrt{H}$. In this regard, matrix 𝒥H𝒥^†^ can be viewed as the product of two positive definite matrices. It is evident that the eigenvalues of 𝒥H𝒥^†^ are greater than zero and det(𝒥H𝒥^†^) = det(𝒥L*L*^T^𝒥^T^)det((𝒥𝒥^T^)−1)>0 with det(·), denoting the determinant of a matrix. According to the properties of the diagonal elements of the matrix, it can be concluded that the diagonal elements of 𝒥H𝒥^†^ are greater than zero (*a*_11_>0, *a*_22_>0). Furthermore, Equation (12) can be rewritten as


$$\begin{array}{l} \left[\begin{array}{c} {\ddot{\xi }}_{1}\\ {\ddot{\xi }}_{2} \end{array}\right]=\left[\begin{array}{cc} a_{11} & a_{12}\\ a_{21} & a_{22} \end{array}\right]\left[\begin{array}{c} -\alpha {\dot{\xi }}_{1}-\beta \delta \xi_{1}-{\left(\dot{J}\dot{\phi }\right)}_{1}\\ -\alpha {\dot{\xi }}_{2}-\beta \xi_{2}-{\left(\dot{J}\dot{\phi }\right)}_{2} \end{array}\right]+\left[\begin{array}{c} {\left(\dot{J}\dot{\phi }\right)}_{1}\\ {\left(\dot{J}\dot{\phi }\right)}_{2} \end{array}\right], \end{array}$$

and further we get


$$\begin{array}{l} {\ddot{\xi }}_{1}+a_{11}\alpha {\dot{\xi }}_{1}+a_{11}\beta \xi_{1}=-a_{12}(\alpha {\dot{\xi }}_{2}+\beta \xi_{2}+{\left(\dot{J}\dot{\phi }\right)}_{2})\\ \;\;\;\;\;\;\;\;\;\;\;\;\;\;\;\;\;\;\;\;\;\;\;\;\;\;\;\;\;\;\;\;\;\;+\;\;(1-a_{11}){\left(\dot{J}\dot{\phi }\right)}_{1} \end{array}$$

and


$$\begin{array}{l} {\ddot{\xi }}_{2}+a_{22}\alpha {\dot{\xi }}_{2}+a_{22}\beta \xi_{2}=-a_{21}(\alpha {\dot{\xi }}_{1}+\beta \xi_{1}+{\left(\dot{J}\dot{\phi }\right)}_{1})\\ \;\;\;\;\;\;\;\;\;\;\;\;\;\;\;\;\;\;\;\;\;\;\;\;\;\;\;\;\;\;\;\;\;\;+\;\;(1-a_{22}){\left(\dot{J}\dot{\phi }\right)}_{2} \end{array}$$

which can be regarded as a perturbed second-order constant coefficient differential equation with respect to ξ. In conclusion, pixel error ξ is able to converge exponentially. To illustrate the steady state of the system (Equation 14), further derivations continue to be given. As the pixel error decreases, all joint properties return to the interior of joint constraints. In this sense, joint properties, i.e., $\ddot{\phi }$, $\dot{\phi }$ and ϕ, are all inside the joint limits with *h*_*i*_ = 1. Therefore, Equation (14) can be reorganized as


(15) $$\begin{array}{l} \ddot{\xi }\left(t\right)+\alpha \dot{\xi }\left(t\right)+\beta \xi \left(t\right)=0. \end{array}$$

It is worth mentioning that Equation (15) can be regarded as a second-order constant coefficient differential equation with regard to ξ. Moreover, the solutions of Equation (15) can be segmented into three subcases on account of different settings of α and β, given the original state ξ(0) = **p**−**p**_d_.

Subcase I: As for α^2^−4β>0, the characteristic roots could be obtained simply as $R_{1}=\left(-\alpha +\sqrt{\alpha^{2}-4\beta }\right)/2$ and $R_{2}=\left(-\alpha -\sqrt{\alpha^{2}-4\beta }\right)/2$ with real number R_1_≠R_2_. Therefore, one can readily deduce


$$\begin{array}{l} \xi \left(t\right)=\xi \left(0\right)\left(D_{1}\text{exp}\left(R_{1}t\right)+D_{2}\text{exp}\left(R_{2}t\right)\right), \end{array}$$

with $D_{1}=\alpha /\left(2\sqrt{\alpha^{2}-4\beta }\right)+1/2$ and $D_{2}=1/2-\alpha /\left(2\sqrt{\alpha^{2}-4\beta }\right)$

Subcase II: As to α^2^−4β = 0, calculating characteristic roots generates R_1_ = R_2_ = −α/2. Hence, it can be readily obtained:


$$\begin{array}{l} \xi \left(t\right)=\xi \left(0\right)\text{exp}\left(-\alpha /2t\right)\left(1+\alpha /2t\right). \end{array}$$

Subcase III: As to α^2^−4β < 0, we get two complex number roots as R_1_ = ζ+*iη* and R_2_ = ζ−*iη*. Accordingly, it is evident that


$$\begin{array}{l} \xi \left(t\right)=\xi \left(0\right)\text{exp}\left(-\zeta t\right)\left(\text{cos}\left(\eta t\right)-\zeta \text{sin}\left(\eta t\right)/\eta \right). \end{array}$$

The above three subcases indicate that the pixel error ξ = **p**−**p**_d_ converges to zero over time globally. The proof is complete.

## 4 DAIVS solution

The existing IBVS schemes, including the AIVS scheme (9), often require a detailed knowledge of the robot visual servoing system. However, in a non-ideal state, many unknown cases often exist, which can disturb the precise control of the robot, thus resulting in large errors. Recalling the data-driven learning algorithm (Equation 8), virtual visual Jacobian matrix $\bar{J}$ is exploited to solve this issue.

### 4.1 DAIVS scheme and RNN solution

Based on the virtual visual Jacobian matrix, a DAIVS scheme (8) would be designed as


$$\begin{array}{l} \text{minimize }\frac{1}{2}{\ddot{\phi }}^{\text{T}}\ddot{\phi }\\ \text{subject to }\ddot{\text{p}}=\bar{J}\ddot{\phi }+\dot{\bar{J}}\dot{\phi }\\ \;\text{p}={\text{p}}_{\text{d}}\\ \;\ddot{\phi }\in \gamma . \end{array}$$

It is a remarkable fact that the DAIVS scheme does not involve the visual structure of the real robot. Instead, the virtual visual Jacobian matrix $\bar{J}$ conveys the transformation relationship between the joint space and image space to deal with possible unknowns in the structure of the robot system. Compared with acceleration-level visual servoing schemes (Keshmiri et al., 2014; Anwar et al., 2019), the proposed DAIVS scheme offers two distinct advantages. First, it prioritizes the safety aspect by considering joint limits. Second, the DAIVS scheme takes into account the uncertainty of the robot vision system and employs the virtual visual Jacobian matrix for robot control, enhancing the fault tolerance ability. The existing acceleration-level visual servoing schemes (Keshmiri et al., 2014; Anwar et al., 2019) cannot accurately implement visual servoing tasks when the Jacobian matrix lacks precision. Furthermore, combining Equations (8, 13) generates


(16a) $$\begin{array}{l} \ddot{\phi }=P_{\gamma }\left({\bar{J}}^{†}\left(-\alpha \dot{\text{p}}-\beta \left(\text{p}-{\text{p}}_{\text{d}}\right)-\dot{\bar{J}}\dot{\phi }\right)\right) \end{array}$$

(16b) $$\begin{array}{l} \dot{\bar{J}}=-\delta \left(\bar{J}\dot{\phi }-\dot{\text{p}}\right){\dot{\phi }}^{\text{T}}. \end{array}$$

It is worth pointing out that the RNN (16) is divided into the inner cycle and outer cycle, i.e., the learning cycle and control cycle. Subsystem (Equation 16a), which can be viewed as the outer cycle, mainly generates the control signal to adjust the joint properties via virtual visual Jacobian matrix $\bar{J}$. In return, inner cycle (Equation 16b) with learning ability can explore the relationship between end-effector motion and joint motion, thus producing virtual visual Jacobian matrix $\bar{J}$ to simulate the movement process of real robots. From a control point of view, the inner cycle (Equation 16b) must converge faster than the outer cycle (Equation 16a). In this sense, δ≫α is a necessary condition for the normal operation of the system.

Note that both RNN (13) and RNN (16) involve the use of pseudo-inverse operations. As a result, various existing methods can be employed to mitigate singularity issues, such as the damped least squares method. Specifically, 𝒥^†^ can be calculated via 𝒥^†^ = 𝒥^T^(𝒥𝒥^T^+*h*I)−1 with *h* being a tiny constant and I being an identity matrix. The additional item *h*I ensures that all eigenvalues of 𝒥𝒥^T^+*h*I are never zero during the inversion process, thereby preventing singular issues. In addition, RNN (16) relies on the virtual visual Jacobian matrix and estimates the real Jacobian matrix using Equation (16b). This enables a robust handling of the visual system's uncertainty. However, RNN (13) relies on the real visual Jacobian matrix, leading to potential inaccuracies in the robot control process.

### 4.2 Stability analyses of RNN

The learning and control performance of the proposed DAIVS scheme aided with RNN (16) are proved by the following theorem.

*Theorem 2:* The Jacobian matrix error $E=\bar{J}-J$ and pixel error ξ = **p**−**p**_d_ produced by RNN (16) converges to zero, given a large enough δ.

*Proof:* The proof is segmented into two parts: (1) proving learning convergence; (2) proving control convergence.

*Part 1:* Proving learning convergence. Design the *i*-th system of Jacobian matrix error as $E_{i}={\bar{J}}_{i}-J_{i}$ (*i* = 1, 2) where ${\bar{J}}_{i}$ and 𝒥_*i*_ denote the *i*-th row of $\bar{J}$ and 𝒥 and set the Lyapunov candidate $V_{i}=\left({\bar{J}}_{i}-J_{i}\right){\left({\bar{J}}_{i}-J_{i}\right)}^{\text{T}}$. Calculating the time derivative of 𝒱_*i*_ leads to


$$\begin{array}{l} {\dot{V}}_{i}=({\dot{\bar{J}}}_{i}-{\dot{J}}_{i})({\bar{J}}_{i}-J_{i}{)}^{\text{T}}\\ \;=-\delta ({\bar{J}}_{i}\dot{\phi }-{\dot{p}}_{i}){\dot{\phi }}^{\text{T}}({\bar{J}}_{i}-J_{i}{)}^{\text{T}}-{\dot{J}}_{i}({\bar{J}}_{i}-J_{i}{)}^{\text{T}}\\ \;=-\delta ({\bar{J}}_{i}\dot{\phi }-J_{i}\dot{\phi }){\dot{\phi }}^{\text{T}}({\bar{J}}_{i}-J_{i}{)}^{\text{T}}-{\dot{J}}_{i}({\bar{J}}_{i}-J_{i}{)}^{\text{T}}\\ \;\leq -\delta \Pi (\dot{\phi }{\dot{\phi }}^{\text{T}})({\bar{J}}_{i}-J_{i})({\bar{J}}_{i}-J_{i}{)}^{\text{T}}-{\dot{J}}_{i}({\bar{J}}_{i}-J_{i}{)}^{\text{T}}, \end{array}$$

where ${\dot{\text{p}}}_{i}$ represents the *i*-th element of $\dot{\text{p}}$, and $\Pi \left(\dot{\phi }{\dot{\phi }}^{\text{T}}\right)$ denotes the least eigenvalue of matrix $\dot{\phi }{\dot{\phi }}^{\text{T}}$. When the manipulator is tracking the feature, the value of $\Pi \left(\dot{\phi }{\dot{\phi }}^{\text{T}}\right)$ is always greater than zero. In this case, we substitute $E_{i}={\bar{J}}_{i}-J_{i}$ into the above equation, resulting in the following expression:


$$\begin{array}{l} {\dot{V}}_{i}\leq -\delta \Pi (\dot{\phi }{\dot{\phi }}^{\text{T}})E_{i}E_{i}^{\text{T}}-{\dot{J}}_{i}E_{i}^{\text{T}}\\ \;\leq -\delta \Pi (\dot{\phi }{\dot{\phi }}^{\text{T}})||E_{i}|{|}_{2}^{2}+||{\dot{J}}_{i}|{|}_{2}||E_{i}|{|}_{2}\\ \;=||E_{i}|{|}_{2}(||{\dot{J}}_{i}|{|}_{2}-\delta \Pi (\dot{\phi }{\dot{\phi }}^{\text{T}})||E_{i}|{|}_{2}). \end{array}$$

For further analysis, we consider three cases based on the above equation:

- If $||E_{i}|{|}_{2}>||{\dot{J}}_{i}|{|}_{2}/\delta \Pi \left(\dot{\phi }{\dot{\phi }}^{\text{T}}\right)$, we observe ${\dot{V}}_{i}<0$ and 𝒱_*i*_>0. This indicates that in this case, *E*_*i*_ converges until $||E_{i}|{|}_{2}=||{\dot{J}}_{i}|{|}_{2}/\delta \Pi \left(\dot{\phi }{\dot{\phi }}^{\text{T}}\right)$.
- If $||E_{i}|{|}_{2}=||{\dot{J}}_{i}|{|}_{2}/\delta \Pi \left(\dot{\phi }{\dot{\phi }}^{\text{T}}\right)$, we find ${\dot{V}}_{i}\leq 0$ and 𝒱_*i*_>0. This implies that *E*_*i*_ will continue to converge or remain at the state with $||E_{i}|{|}_{2}=||{\dot{J}}_{i}|{|}_{2}/\delta \Pi \left(\dot{\phi }{\dot{\phi }}^{\text{T}}\right)$.
- If $||E_{i}|{|}_{2}<||{\dot{J}}_{i}|{|}_{2}/\delta \Pi \left(\dot{\phi }{\dot{\phi }}^{\text{T}}\right)$, we have two possibilities: either ${\dot{V}}_{i}>0$ and 𝒱_*i*_> 0, or ${\dot{V}}_{i}\leq 0$ and 𝒱_*i*_> 0. In the former possibility, the error will increase until ||*E*_*i*_||_2_= $||{\dot{J}}_{i}|{|}_{2}/\delta \Pi \left(\dot{\phi }{\dot{\phi }}^{\text{T}}\right)$. In the latter possibility, the error will continue to converge or remain constant.

Combining the above three cases, it can be summarized that $\underset{t\to +\infty }{\lim}||E_{i}|{|}_{2}\leq ||{\overset{{}^{\circ}}{J}}_{i}|{|}_{2}/\delta \Pi \left(\overset{{}^{\circ}}{\phi }{\overset{{}^{\circ}}{\phi }}^{\text{T}}\right)$. Furthermore, it can be deduced that the Jacobian matrix error $E=\bar{J}-J$ produced by RNN (16a) globally approach zero, given a sufficiently large value of δ.

*Part 2:* Proving control convergence.

According to the proof in *Part 1*, we take advantage of the LaSalle's invariant principle (Khalil, 2001) again to conduct the convergence proof on Equation (16b). In other words, the following formula is provided by replacing $\bar{J}$ and $\dot{\bar{J}}$ with 𝒥 and $\dot{J}$:


(17) $$\begin{array}{l} \ddot{\phi }=P_{\gamma }\left(J^{†}\left(-\alpha \dot{\text{p}}-\beta \left(\text{p}-{\text{p}}_{\text{d}}\right)-\dot{J}\dot{\phi }\right)\right), \end{array}$$

which is equivalent to Equation (13). In consequence, the proof on the convergence of the pixel error **p**−**p**_d_ in Equation (17) has been discussed in Theorem 1 and thus omitted here. The proof is complete.

## 5 Simulation verifications

In this section, simulations are conducted on a Franka Emika Panda manipulator with 7 DOFs for completing a visual servoing task, which are synthesized by the proposed AIVS scheme (9) and the proposed DAIVS scheme. Note that the AIVS scheme (9) is able to drive the redundant manipulator to perform the visual servoing task with a given visual Jacobian matrix, and that, the DAIVS scheme can deal with the unknown situation in the robot system dynamically in the absence of the visual Jacobian matrix. For the simulations, this study utilizes a computer with an Intel Core i7-12700 processor and 32 GB RAM. The simulations are performed using MATLAB/Simulink software version R2022a.

First, some necessary information and parameter settings about the manipulator and camera structure are given below. The Franka Emika Panda manipulator is a 7-DOF redundant manipulator (Gaz et al., 2019), with a camera mounted on its end-effector. In addition, we set *l* = 8 × 10^−3^ m, *u*_0_ = *v*_0_ = 256 pixel, $a_{x}=a_{y}=8\times 10^{4}\;\text{pixel/m}$, and design μ = ν = 20 with *z* = 2, task execution time *T* = 20 s and ${\text{p}}_{\text{d}}={\left[256,256\right]}^{\text{T}}$ pixel. In addition, the joint limits are set as ${\ddot{\phi }}^{+}=-{\ddot{\phi }}^{-}={\left[2\right]}_{7\times 1}$ rad/s^2^, ${\dot{\phi }}^{+}=-{\dot{\phi }}^{-}={\left[0.6\right]}_{7\times 1}$ rad/s, $\phi^{+}=-\phi^{-}={\left[2.5\right]}_{7\times 1}$ rad and θ = [0.076]_7 × 1_ rad. It is noteworthy that the parameters can be divided into two categories: structural parameters and convergence parameters. Structural parameters, such as *l*, *u*_0_, *v*_0_, *a*_*x*_, and *a*_*y*_, are dependent on the configuration of the visual servo system. On the other hand, the convergence parameters, namely, μ, ν, α, β, and δ, play a vital role in adjusting the convergence behavior of RNN (16). These convergence parameters are set to values greater than zero, and their specific values can be determined through the trial and error method.

### 5.1 Simulation of AIVS scheme

**Figure 3:**
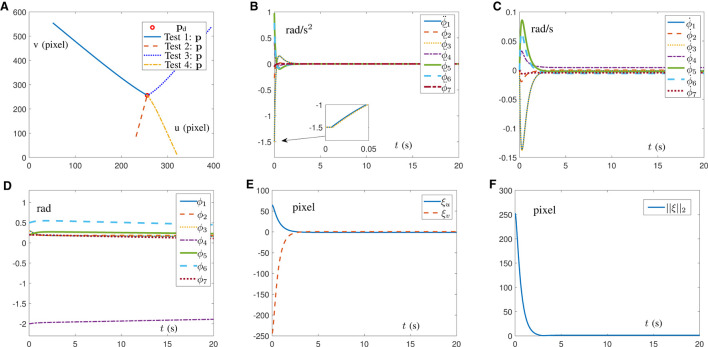
Simulations on a Franka Emika Panda manipulator carrying out IBVS task synthesized by the AIVS scheme (9) assisted by RNN (13) with four test examples. **(A)** Profiles of feature trajectories and desired pixel point in four tests. **(B)** Profiles of joint acceleration in test 4. **(C)** Profiles of joint velocity in test 4. **(D)** Profiles of joint angle in test 4. **(E)** Profiles of pixel error in test 4. **(F)** Profiles of Euclidean norm of pixel error in test 4.

In this subsection, in order to prove the feasibility of the AIVS scheme (9), four simulations with different initial position states of the Franka Emika Panda manipulator are conducted to trace one desired feature with results shown in Figure 3. Simply design α = 10 and β = 10. It would be readily discovered from Figure 3A that four test examples from four different directions are straightforward to successfully pursue the desired pixel. With test 4 as an example, detailed joint data and pixel errors are shown in Figure 3B through Figure 3F, which illustrate that the joint angle, joint velocity, and joint acceleration are all kept inside the joint limit and that the pixel error can converge to zero within 5 s. The above descriptions well verify the validity of the proposed AIVS scheme (9) in the case of the known visual servoing Jacobian matrix to solve the visual servoing problem at the acceleration level.

### 5.2 Simulation of DAIVS scheme

**Figure 4:**
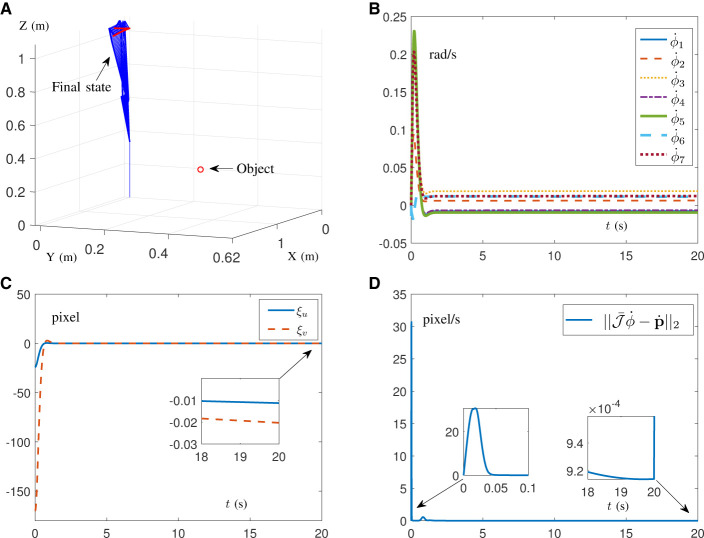
Simulation results on a Franka Emika Panda manipulator with unknown structure carrying out the IBVS task synthesized by the DAIVS scheme assisted with RNN (16). **(A)** Profiles of the movement process. **(B)** Profiles of the joint acceleration. **(C)** Profiles of the pixel error. **(D)** Profiles of the Euclidean norm of learning error.

This subsection indicates the feasibility and capability of the pixel error convergence of the DAIVS scheme aided with the RNN (16) by providing simulation results, as shown in Figure 4. Furthermore, we choose δ = 2 × 10^4^, α = 10 and β = 40. Notably, the virtual visual Jacobian matrix is exploited with random initial values, instead of the real visual Jacobian matrix to facilitate system operation. The end-effector of the robotic arm is oriented toward the object, as shown in Figure 4A. In addition, the joint acceleration is shown in Figure 4B, which is confined to the joint limit and maintain the normal operation. As shown in Figure 4C, the Franka Emika Panda manipulator successfully traces the desired feature with pixel error converging to zero and maintaining the order of 10^−2^ pixel. As for the learning ability, Figure 4D illustrates that the virtual robot manipulator can learn the movement of the real robot manipulator with the learning error approaching to zero in 0.05 s and maintaining the order of 10^−4^ pixel/s. In short, the simulation results in Figure 4 highlight the simultaneous learning and control ability of RNN (16).

### 5.3 Comparisons of proposed schemes

This subsection offers simulation comparison results between the proposed schemes aided with the corresponding RNNs and the IBVS method presented in the study by Zhang and Li (2018). In this regard, the RNN provided in the study by Zhang and Li (2018) is shown as


(18) $$\dot{\phi }=P_{\gamma }(-\kappa_{1}J^{\text{T}}(p-p_{\text{d}})-\kappa_{2}J^{\text{T}}\int_{0}^{t}\left(p-p_{\text{d}}\right)\text{d}t),$$

where parameters κ_1_>0 and κ_2_>0 determine the rate of error convergence. It is worth pointing out that the IBVS method in the study by Zhang and Li (2018) assisted with RNN (18) is constructed from the viewpoint of the velocity level, and that, RNN (18) requires exact structural information 𝒥 to maintain the normal operation.

**Figure 5:**
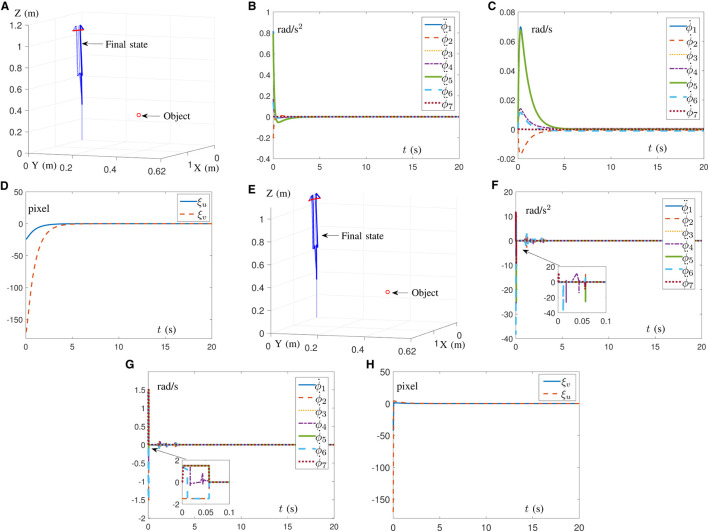
Simulation results on a Franka Emika Panda manipulator with accurate structure information carrying out IBVS task. **(A)** Profiles of motion process assisted with RNN (13). **(B)** Profiles of joint acceleration assisted with RNN (13). **(C)** Profiles of joint velocity assisted with RNN (13). **(D)** Profiles of pixel error assisted with RNN (13). **(E)** Profiles of motion process assisted with RNN (18). **(F)** Profiles of joint acceleration assisted with RNN (18). **(G)** Profiles of joint velocity assisted with RNN (18). **(H)** Profiles of pixel error assisted with RNN (18).

In the first place, simulations are conducted on the Franka Emika Panda manipulator for IBVS task with Figures 5A–D synthesized by RNN (13) and Figures 5E–H synthesized by RNN (18). Notably, the results in Figure 5 are carried out on the premise of known structural information 𝒥 with parameters κ_1_ = κ_2_ = 2, α = 10, and β = 10. As shown in Figures 5A, E, the manipulator's end-effector is controlled to point toward the object. In Figure 5B, the joint acceleration generated by RNN (13) is safely confined within the joint limits, while the joint acceleration generated by RNN (18) exists a sudden change of ~38 rad/s^2^ in Figure 5F, which may cause damage to the robot. Furthermore, in contrast to Figure 5G, the joint velocity shown in Figure 5C is smaller and exhibits smoother changes, making it more suitable for real-world scenarios. Figures 5D, H demonstrate that both RNN (13) and RNN (18) are able to quickly propel pixel errors to zero. Therefore, it is concluded from the above results that AIVS scheme (9) aided by RNN (13) is able to guarantee a better safety performance when controlling the manipulator.

**Figure 6:**
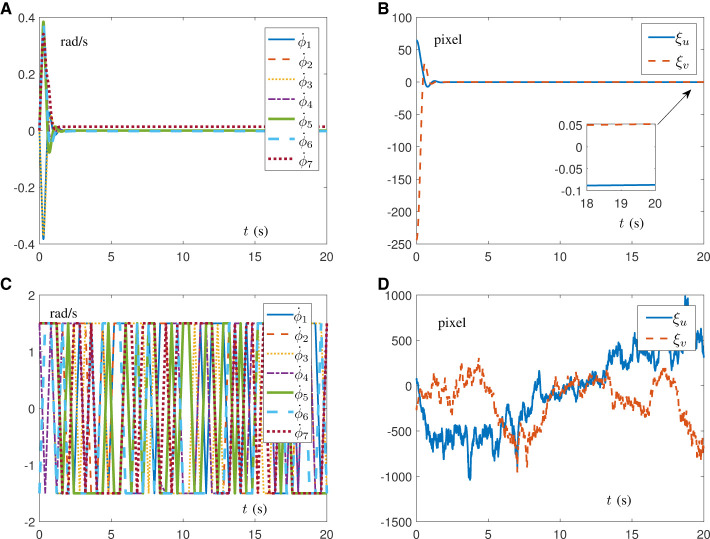
Simulation results on a Franka Emika Panda manipulator carrying out IBVS task with unknown structure. **(A)** Profiles of joint velocity assisted with RNN (16). **(B)** Profiles of pixel error assisted with RNN (16). **(C)** Profiles of joint velocity assisted with RNN (18). **(D)** Profiles of pixel error assisted with RNN (18).

Beyond that, in the case of the unknown visual system, corresponding comparison simulations are driven by the DAIVS scheme aided with the RNN (16) and the IBVS method in the study by Zhang and Li (2018) assisted with RNN (18). The results are shown in Figure 6 with parameters κ_1_ = κ_2_ = 2, α = 10, β = 40, and δ = 2 × 10^4^. To simulate the unknown visual system, $\bar{J}$ in Equation (16) and 𝒥 in Equation (18) are random matrices of constants with the absolute value of each element < 100. Figures 6A, B well embody that, when encountering unknown structural information, the DAIVS scheme assisted with RNN (16) controls the Franka Emika Panda manipulator to preferably complete IBVS task with the pixel error converging to zero. Nevertheless, the generated joint velocity in Figure 6C changes dramatically within the joint limit in a mess. Even worse, the pixel error driven by RNN (18) does not converge and maintain a diffused state, which indicates the failure of the IBVS task. In conclusion, the proposed DAIVS scheme is able to deal with the unknown structural information in the robot system and fulfill the visual servo control with simultaneous learning and control performance.

Furthermore, comparison results among different existing approaches (Hashimoto et al., 1991; Keshmiri et al., 2014; Zhang et al., 2017; Van et al., 2018; Zhang and Li, 2018; Anwar et al., 2019; Li et al., 2020; Zhu et al., 2022) for visual servoing of robot manipulators are presented in Table 1. It is worth emphasizing that, compared with the prior art, the proposed RNN (13) and RNN (16) are the first acceleration-level work, considering the multiple levels of joint constraints, and RNN (16) is the first study to dispose the unknown situations in the robot visual system with simultaneous learning and control ability. As a result, the above two points are the innovative contributions of this study.

**Table 1:** Comparisons among different approaches for visual servoing of robot manipulators.

	**Visual**	**Scheme**	**Velocity**	**Acceleration**	**Structure**	**Jacobian matrix**
	**servoing**	**level**	**constraints**	**constraints**	**information**	**learning**
RNN (13)	Yes	Acceleration	Yes	Yes	Unnecessary	Yes
RNN (16)	Yes	Acceleration	Yes	Yes	Necessary	No
Van et al. (2018)	Yes	Velocity	No	No	Necessary	No
Hashimoto et al. (1991)	Yes	Velocity	No	No	Necessary	No
Zhang et al. (2017)	Yes	Velocity	Yes	No	Necessary	No
Zhang and Li (2018)	Yes	Velocity	Yes	No	Necessary	No
Li et al. (2020)	Yes	Velocity	Yes	No	Necessary	No
Keshmiri et al. (2014)	Yes	Acceleration	No	No	Necessary	No
Anwar et al. (2019)	Yes	Acceleration	No	No	Necessary	No
Zhu et al. (2022)	Yes	Torque	No	No	Necessary	No

## 6 Experiments on real manipulators

To verify the effectiveness and practicability of the proposed DAIVS scheme, physical experiments on a real manipulator are conducted in this section, which are driven by the DAIVS scheme aided with RNN (16). Specifically, the experiments essentially rely on C++ and the visual servoing platform (ViSP) for embedding algorithms and control (Marchand et al., 2005), which are built on ubuntu 16.04 LTS operating system. In addition, the experiment platform consists of a Franka Emika Panda manipulator, an Intel RealSense Camera D435i, a personal computer, and an AprilTag (target). It is worth mentioning that the acceleration control commands generated by the proposed RNN (16) are transmitted in a discrete form with a frequency of 1,000 Hz, and parameter settings of RNN (16) are designed as follows. We choose α = 10, β = 10, δ = 10^6^, μ = ν = 20, ${\ddot{\phi }}^{+}=-{\ddot{\phi }}^{-}={\left[15,\;7.5,\;10,\;12.5,\;15,\;20,\;20\right]}^{\text{T}}$ rad/s^2^, ${\dot{\phi }}^{+}=-{\dot{\phi }}^{-}={\left[2.1,2.1,2.1,2.1,2.6,2.6,2.6\right]}^{\text{T}}$ rad/s, ϕ^+^ = [2.8, 1.7, 2.8, −0.1, 2.9, 3.7, 2.8]^T^ rad, ϕ^−^ = [−2.8, −1.7, −2.8, −3.0, −2.8, −0, −2.8]^T^ rad, and $\bar{J}\left(0\right)=J\left(0\right)$. As for the parameter settings of the camera and pixel coordinates, they can be directly referenced to ViSP (Marchand et al., 2005). Different from the previous simulations, the physics experiments set the target as an AprilTag containing four features. As a result, the physical parameters associated with the features are expanded to 8 instead of 2.

**Figure 7:**
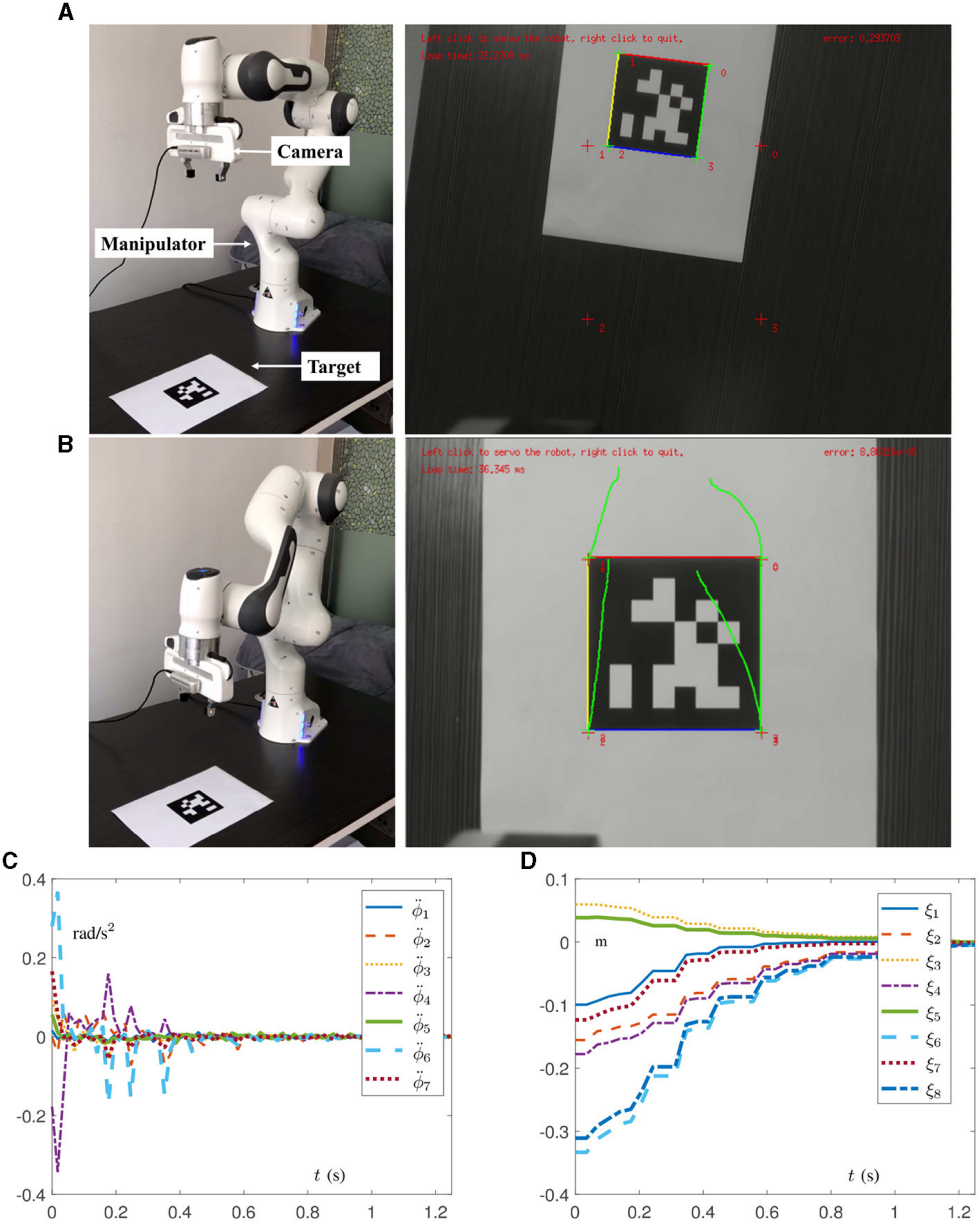
Physical experiments on a Franka Emika Panda manipulator assisted with RNN (16) for carrying out IBVS task with a fixed target. **(A)** Initial states of the manipulator and camera. **(B)** Final states of the manipulator and camera. **(C)** Profiles of joint acceleration. **(D)** Profiles of tracking error.

**Figure 8:**
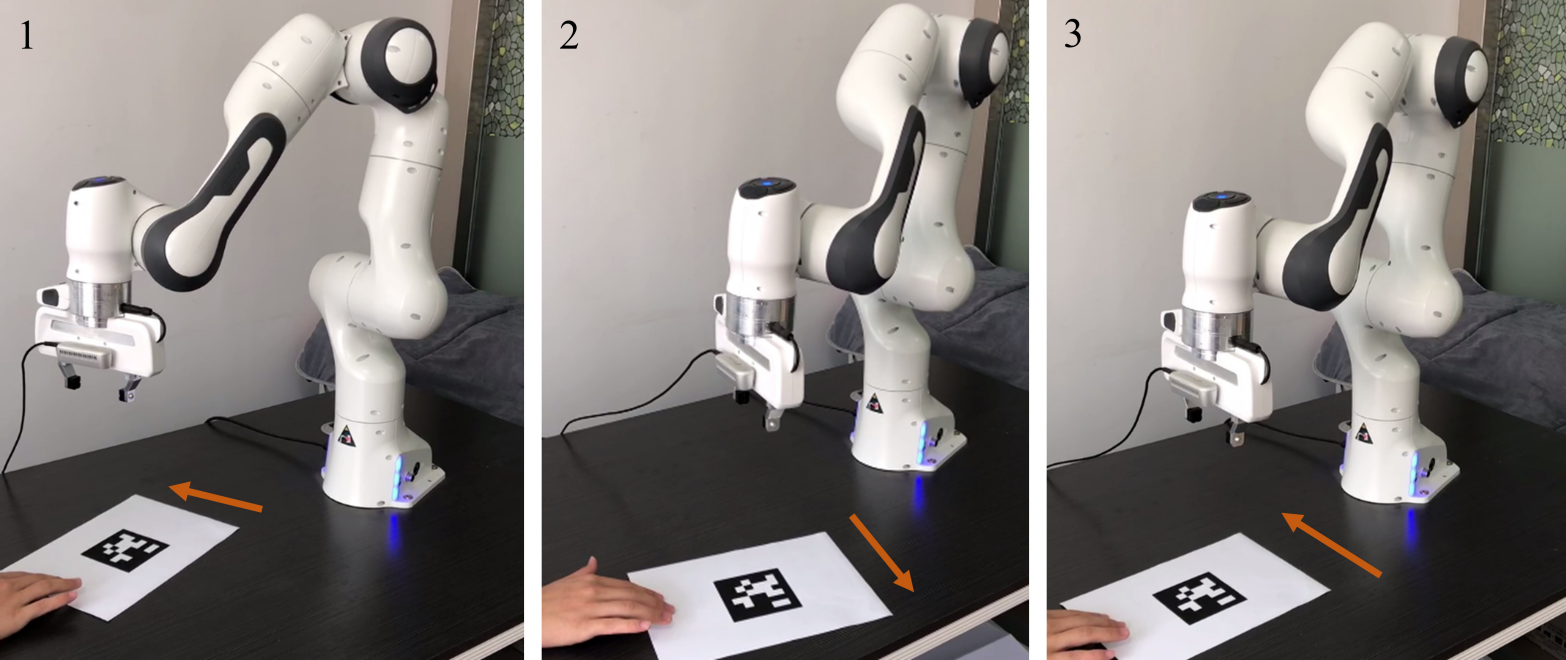
Physical experiments on a Franka Emika Panda manipulator assisted with RNN (16) for carrying out IBVS task with a moving target.

Experiment results on the Franka Emika Panda manipulator tracking the fixed target are shown in Figures 7, 8 with ${\text{p}}_{\text{d}}={\left[-0.06,\;-0.06,\;0.06,\;-0.06,\;0.06,\;0.06,\;-0.06,\;0.06\right]}^{\text{T}}$ m for the given task in the camera system. It is worth mentioning that the robot manipulator adjusts the joint state to recognize and approach the target, and when the pixel error reaches the order of 10^−5^ pixel, the task automatically completes. It is important that the whole process of learning and control does not involve the real Jacobian matrix to simulate the situation of the unknown structure. In Figures 7A, B, the initial and final states of the manipulator and camera indicate that the visual servoing task is successfully realized by the DAIVS scheme with execution time of 1.25 s. Specifically, the joint acceleration in Figure 7C varies normally within the joint constraints. In the meantime, the tracking errors ξ of four features are presented in Figure 7D, which illustrate the precise control ability of the DAIVS scheme with global convergence to zero.

Beyond that, experiments on the Franka Emika Panda manipulator tracking the moving target are conducted to demonstrate the feasibility of the DAIVS scheme. In Figure 8, the AprilTag is moved artificially by the hand toward the left and right and simultaneously the manipulator constantly adjusts joint states to achieve the characteristics of real-time visual tracking. More vividly, the experiment videos corresponding to Figures 7, 8 are available at https://youtu.be/6uw35bidVcw.

## 7 Conclusion

This study has proposed an AIVS scheme for robot manipulators, taking into account joint limits at multiple levels. On this basis, incorporating data-driven techniques, a DAIVS scheme has been proposed to handle potential unknown situations in the robot visual system. Furthermore, RNNs have been exploited to generate the online solution corresponding to the proposed schemes with theoretical analyses, demonstrating the simultaneous learning and control ability of the proposed DAIVS scheme. Then, numerous simulations and experiments have been carried out on a Franka Emika Panda manipulator to track the desired feature. The results validate the theoretical analyses, demonstrate the feasibility of the AIVS scheme, and showcase the fast convergence and robustness of the DAIVS scheme. Compared with the method in the study by Zhang and Li (2018), the DAIVS scheme exhibits superior learning capability and achieves visual servoing control with the unknown Jacobian matrix.

In summary, this study provides a data-driven approach for the precise manipulation of robots in IBVS tasks, addressing unknown situations that could affect the robot's Jacobian matrix. In the future, we aim to expand our research to incorporate dynamic factors, utilizing joint torque as control signals and considering dynamic uncertainties.

## Data availability statement

The original contributions presented in the study are included in the article/supplementary material, further inquiries can be directed to the corresponding author.

## Author contributions

LW: Investigation, Methodology, Resources, Software, Writing – original draft. ZX: Data curation, Investigation, Writing – review & editing.
